# Supplementary material for: Capturing the blue-light activated state of the Phot-LOV1 domain from Chlamydomonas reinhardtii using time-resolved serial synchrotron crystallography
Source: IUCrJ. 2024 Jul 22;11(Pt 5):792–808. doi: 10.1107/S2052252524005608 (PMC11364019; doi:10.1107/S2052252524005608)
Supplement: Supplementary file 1 [file m-11-00792-sup1.pdf]

IUCrJ

Volume 11 (2024)

Supporting information for article:

Capturing the blue-light activated state of the Phot-LOV1 domain from *Chlamydomonas reinhardtii* using time-resolved serial synchrotron crystallography

Guillaume Gotthard, Sandra Mous, Tobias Weinert, Raiza Nara Antonelli Maia, Daniel James, Florian Dworkowski, Dardan Gashi, Antonia Furrer, Dmitry Ozerov, Ezequiel Panepucci, Meitian Wang, Gebhard F. X. Schertler, Joachim Heberle, Joerg Standfuss and Przemyslaw Nogly

Contents

Supplementary Tables and Figures page 2

Supplementary References page 9

Supplementary materials

Supplementary Tables

Table S1 Structures of LOV domains in the Protein Data Bank

Protein origin		Experimental details						References
Name	Organism	State	Resolution	Dose (kGy)	Timing (ms)	DC T	PDB ID	Associated publication
Phy1 LOV2	*Avena sativa*	dark	1.4			105	2V0U	(Halavaty & Moffat, 2007)
Phy1 LOV2	*Avena sativa*	light	1.7			105	2V0W	
Phy1 LOV2	*Avena sativa*	dark	1.65			293	2V1A	
Phy1 LOV2	*Avena sativa*	light	1.55			293	2V1B	
LOV2-Zdk1	*Avena sativa*	dark	2.1			100	5EFW	(Wang *et al.*, 2016)
LOV2-Zdk2-C450A	*Avena sativa*	dark	1.4			100	5DJT	
LOV2-Zdk3-C450A	*Avena sativa*	dark	2.1			100	5DJU	
PA-Rac1	*Avena sativa*	dark	1.9			100	2WKP	(Wu *et al.*, 2009)
PA-Rac1-C450A	*Avena sativa*	dark	1.6			100	2WKQ	
PA-Rac1-C450M	*Avena sativa*	dark	2.2			100	2WKR	
Phy2 LOV2	*Arabidopsis thaliana*	dark	1.7			100	4EEP	(Christie *et al.*, 2012)
Phy2 LOV2-C426A	*Arabidopsis thaliana*	dark	1.75			100	4EER	
Phy2 LOV2	*Arabidopsis thaliana*	light	1.7	24		100	6QQI	(Gotthard *et al.*, 2019)
Phy2 LOV2	*Arabidopsis thaliana*	dark	1.38	2680		100	6QQH	
Phy2 LOV2	*Arabidopsis thaliana*	light	1.7	48		100	6QSA	
Phy2 LOV2	*Arabidopsis thaliana*	light	2.4	34		293	6QQK	
Phy2 LOV2	*Arabidopsis thaliana*	dark	2.08	354		293	6QQJ	
Phy2 LOV2	*Arabidopsis thaliana*	dark	2.2		0	293	6S45	(Aumonier *et al.*, 2020)
Phy2 LOV2	*Arabidopsis thaliana*	light	2.75		4158	293	6S46	
Phy1 LOV2	*Arabidopsis thaliana*	dark	2.75			105	4HHD	(Halavaty & Moffat, 2013)
iLOV	*Arabidopsis thaliana*	dark	1.8			100	4EES	(Christie *et al.*, 2012)
iLOV	*Arabidopsis thaliana*	dark	1.2			100	4EET	
iLOV-Q489K	*Arabidopsis thaliana*	dark	1.45			100	7ABY	(Röllen *et al.*, 2021)
phiLOV2.1	*Arabidopsis thaliana*	dark	1.41			100	4EEU	(Christie *et al.*, 2012)
Phy2 LOV1	*Arabidopsis thaliana*	dark	2			110	2Z6D	(Nakasako *et al.*, 2008)
Phy1 LOV1	*Arabidopsis thaliana*	dark	2.1			100	2Z6C	
ZTL_LOV	*Arabidopsis thaliana*	dark	2.5			80	5SVG	(Pudasaini *et al.*, 2017)
ZTL_LOV-G80R	*Arabidopsis thaliana*	dark	2.6			80	5SVU	
ZTL_LOV-V48I:G80R	*Arabidopsis thaliana*	dark	2.1			80	5SVV	
ZTL_LOV-V48I:G80R	*Arabidopsis thaliana*	light	2.29			80	5SVW	
ZTL_LOV-G46A:G80R	*Arabidopsis thaliana*	dark	3			100	6WLE	(Pudasaini *et al.*, 2021)
ZTL_LOV-G46S:G80R	*Arabidopsis thaliana*	dark	3			100	6WLP	
Phy3 LOV2	*Adiantum capillus-veneris*	light	2.6			296	1JNU	(Crosson & Moffat, 2002)
Phy3 LOV2	*Adiantum capillus-veneris*	dark	2.73			298	1G28	(Crosson & Moffat, 2001)
Phot1-LOV1	*Chlamydomonas reinhardtii*	dark	1.9			100	1N9L	(Fedorov *et al.*, 2003)
Phot1-LOV1	*Chlamydomonas reinhardtii*	light	2.3			100	1N9N	
Phot1-LOV1	*Chlamydomonas reinhardtii*	light	2.8			100	1N9O	
PtAu1a_LOV	*Phaeodactylum tricornutum*	dark	2.5			100	5DKK	(Heintz & Schlichting, 2016)
PtAu1a_LOV	*Phaeodactylum tricornutum*	light	2.7			100	5DKL	
Aureochrome1 LOV	*Vaucheria frigida*	dark	2.75			100	3UE6	(Mitra *et al.*, 2012)
Aureochrome1 LOV	*Vaucheria frigida*	light	2.9			100	3ULF	
Aureochrome1a LOV	*Phaedodactylum tricornutum*	dark	2.79			100	5A8B	(Banerjee *et al.*, 2016)
Aureochrome1a LOV	*Phaedodactylum tricornutum*	dark	3.44			100	6T73	(Hepp *et al.*, 2020)
Aureochrome1a LOV	*Phaedodactylum tricornutum*	dark	1.9			100	6T74	
Aureochrome1-like	Ochromonas danica	dark	1.66			100	6I22	(Kalvaitis *et al.*, 2019)
Aureochrome1-like	Ochromonas danica	dark	1.5			100	6I21	
Aureochrome1-like	Ochromonas danica	dark	1.43			100	6I24	
Aureochrome1-like	Ochromonas danica	dark	2			100	6I23	
Aureochrome1-like	Ochromonas danica	dark	1.97			100	6I25	
Aureochrome1-like	Ochromonas danica	dark	1.37			100	6I20	
Vivid	*Neurospora crassa*	dark	2			100	2PD7	(Zoltowski *et al.*, 2007)
Vivid-C71S	*Neurospora crassa*	dark	1.8			100	2PD8	
Vivid	*Neurospora crassa*	light	1.7			100	2PDR	
Vivid	*Neurospora crassa*					100	2PDT	
Vivid	*Neurospora crassa*	dark	2.1			100	6CNY	
Vivid	*Neurospora crassa*	light	2.75			100	3RH8	(Vaidya *et al.*, 2011)
Vivid	*Neurospora crassa*	light	2.3			77	3IS2	(Lamb *et al.*, 2009)
Vivid-I74V:I85V	*Neurospora crassa*	dark	1.8			80	3HJI	(Zoltowski *et al.*, 2009)
Vivid-I74V	*Neurospora crassa*	dark	2			80	3HJK	

Vivid-C71V	*Neurospora crassa*	dark	1.65	77	3D72	(Zoltowski & Crane, 2008)
Env1	*Trichoderma reesei*	dark	2.23	100	4WUJ	(Lokhandwala *et al.*, 2015)
DsLOV	*Dinoroseobacter shibae*	dark	1.5	100	4KUK	(Endres *et al.*, 2015)
DsLOV	*Dinoroseobacter shibae*	light	2	100	4KUO	
DsLOV-M49A	*Dinoroseobacter shibae*	dark	1.9	100	6GBA	
DsLOV-M49I	*Dinoroseobacter shibae*	dark	1.86	100	6GAY	(Fettweiss *et al.*, 2018)
DsLOV-M49S	*Dinoroseobacter shibae*	dark	1.752	100	6GB3	
DsLOV-M49T	*Dinoroseobacter shibae*	dark	1.63	100	6GBV	
HK_LOV	*Erythrobacter litoralis*	dark	1.6	100	4R38	(Rivera-Cancel *et al.*, 2014)
HK_LOV	*Erythrobacter litoralis*	dark	2.92	100	4R3A	
LOV_HTH	*Erythrobacter litoralis*	dark	2.1	100	3P7N	(Nash *et al.*, 2011)
LOV-HK	*Brucella melitensis*	dark	1.64	100	3T50	(Rinaldi *et al.*, 2012)
LOV-HK-C69S	*Brucella abortus*	dark	2.34	100	6PH2	
LOV-PAS	*Brucella abortus*	dark	2.74	100	6PH3	(Rinaldi *et al.*, 2021)
LOV-PAS-HK	*Brucella abortus*	light	3.25	100	6PH4	
LOV-PAS	*Brucella abortus*	light	2.8	100	6PPS	
YtvA	*Bacillus subtilis*	light	1.95	100	2PR6	(Möglich & Moffat, 2007)
YtvA	*Bacillus subtilis*	dark	1.45	100	2PR5	
fixL	*Bacillus subtilis*	dark	2.3	100	4GCZ	(Diensthuber *et al.*, 2013)
RsLOV-L32V	*Rhodobacter spheroides*	dark	1.95	100	4HIA	
RsLOV-A138Y	*Rhodobacter spheroides*	dark	2.64	100	4HJ3	
RsLOV	*Rhodobacter spheroides*	dark	2.7	100	4HJ4	(Conrad *et al.*, 2013)
RsLOV	*Rhodobacter spheroides*	dark	2.2	100	4HJ6	
RsLOV	*Rhodobacter spheroides*	light	2.34	100	4HNB	
RsLOV-D109G	*Rhodobacter spheroides*	dark	2	100	7OBZ	(Dietler *et al.*, 2021)
RsLOV-d2	*Rhodobacter spheroides*	dark	1.9	100	7OB0	
W619_1-LOV	*Pseudomonas putida*	apo	2.5	100	5LUV	(Arinkin *et al.*, 2017)
PpSB1-LOV	*Pseudomonas putida*	light	2.63	100	3SW1	(Circolone *et al.*, 2012)
PpSB1-LOV	*Pseudomonas putida*	dark	2.55	100	5J3W	(Röllen *et al.*, 2016)
PpSB1-LOV	*Pseudomonas putida*	light	2.67	100	5J4E	
PpSB1-LOV-R61H:R66I	*Pseudomonas putida*	dark	2.04	100	6GG9	(Fettweiss *et al.*, 2018)
PpSB2-LOV	*Pseudomonas putida*	dark	1.93	100	7A6P	(Arinkin *et al.*, 2021)
CagFbFP	*Chloroflexus aggregans*	dark	1.22	100	6RHG	(Nazarenko *et al.*, 2019)
CagFbFP-C85A	*Chloroflexus aggregans*	dark	1.07	100	6RHF	
CagFbFP-C85A:A56P	*Chloroflexus aggregans*	dark	1.6	100	6Y7R	(Remeeva *et al.*, 2020)
CagFbFP-C85A:A95P	*Chloroflexus aggregans*	dark	1.6	100	6Y7U	
CagFbFP-Q148N	*Chloroflexus aggregans*	dark	1.45	100	6YWG	(Remeeva *et al.*, 2021)
CagFbFP-Q148H	*Chloroflexus aggregans*	dark	1.27	100	6YWQ	
CagFbFP-Q148K	*Chloroflexus aggregans*	dark	1.5	100	6YX6	
CagFbFP-Q148K	*Chloroflexus aggregans*	dark	1.5	100	6YXB	
CagFbFP-Q148K	*Chloroflexus aggregans*	dark	1.36	100	6YX4	(Röllen *et al.*, 2021)
CagFbFP-I52T:Q148K	*Chloroflexus aggregans*	dark	1.8	100	7AB7	
CagFbFP-I52T	*Chloroflexus aggregans*	dark	1.9	100	7AB6	
CisFbFP-C85A	*Chloroflexus islandicus*	dark	1.2	100	7OO9	(Goncharov *et al.*, 2021)
NifL	Azotobacter vinelandii	dark	1.04	100	2GJ3	(Key *et al.*, 2007)

Supplementary Figures

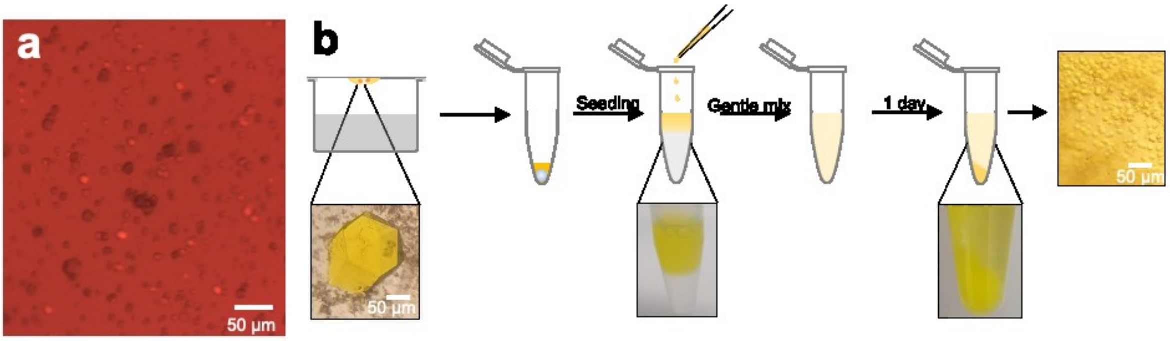

Fig. S1 Crystallization and preparation of *Cr*PhotLOV1 microcrystals. (**a**) Microcrystals obtained in crystallization screening observed under the microscope with a red filter after one day. (**b**) Protocol for preparation of *Cr*PhotLOV1 microcrystals with the batch crystallization method, *i*) macrocrystals are grown with the hanging drop crystallization method, *ii*) crystal seeds are then prepared from macrocrystals with Hampton research seeding beads and *iii*) are mixed with protein solution which is added dropwise into an Eppendorf tube containing the crystallization condition, *iv*) after a gentle mixing by inverting the tube several times, and *v*) one day at room temperature, *vi*) microcrystals appears and sediment at the bottom of the tube.

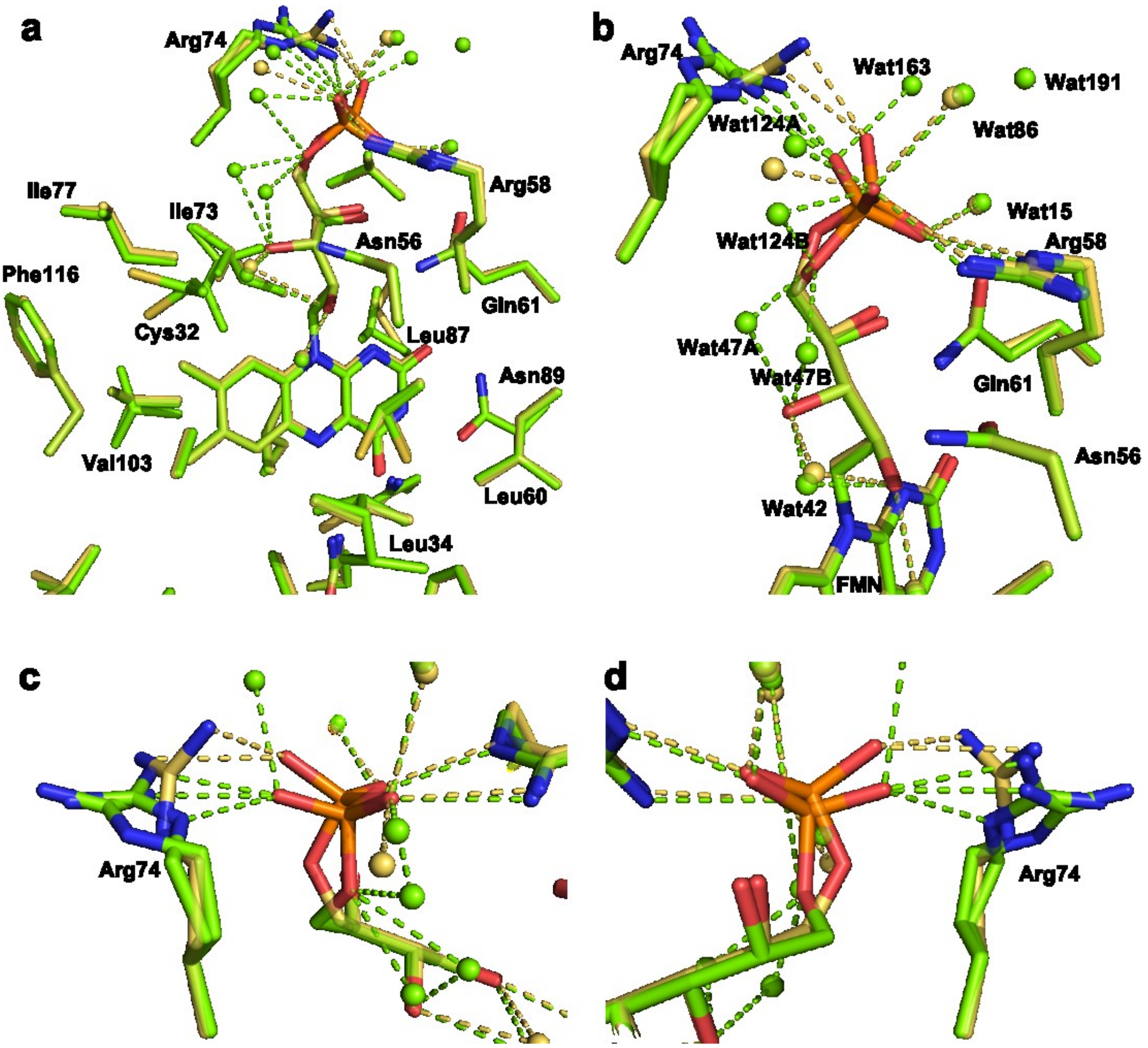

Fig. S2 Differences in the coordination net around the FMN in *Cr*PhotLOV1 dark-state structures at cryogenic temperature. The structure solved in this work (green) is superimposed on the structure 1N9L (transparent yellow) (**a**) far view of the FMN environment, (b) close up on the phosphoribityl tail, (c) and (d) close-up view on the Arg74.

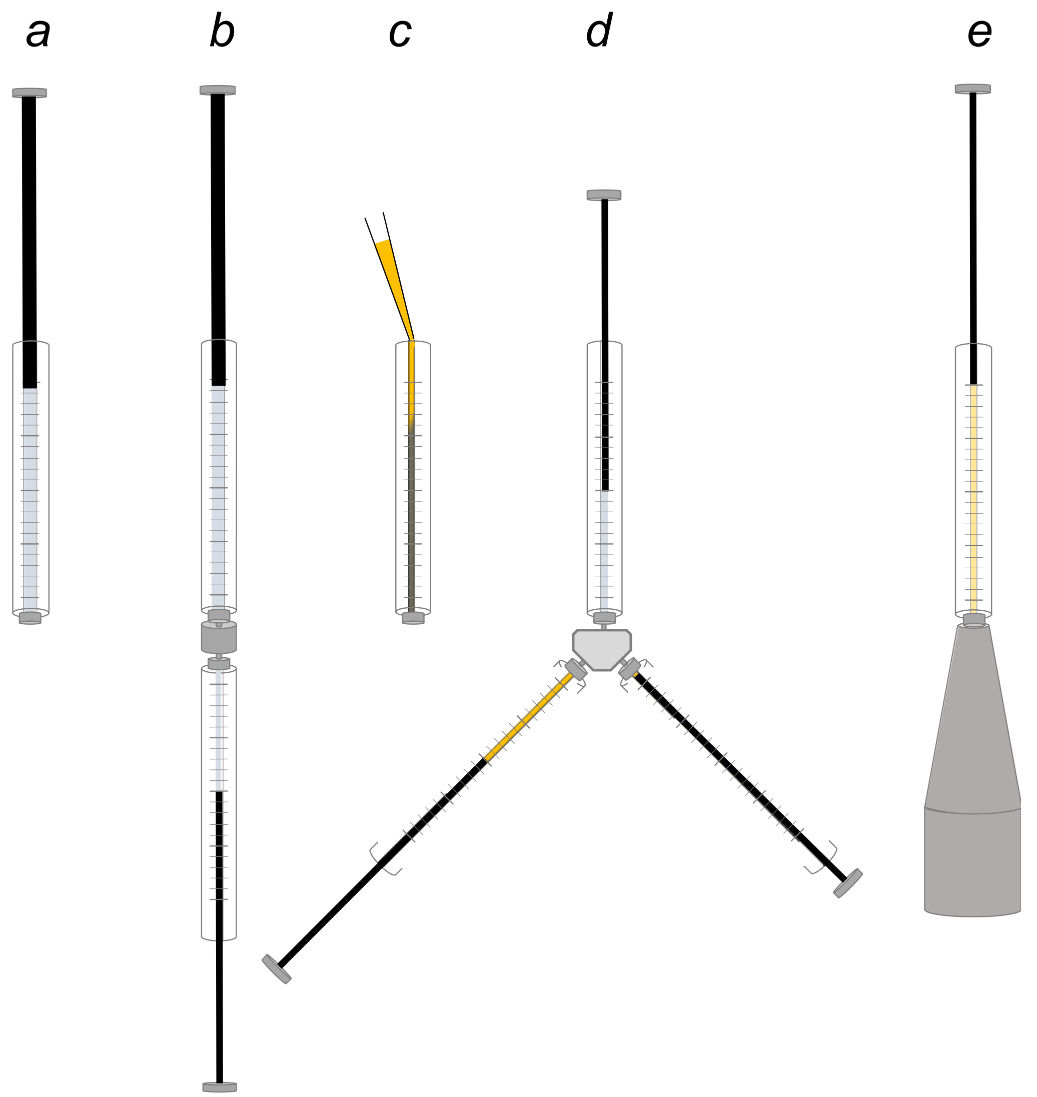

Fig. S3 Protocol for embedding *Cr*PhotLOV1 crystals in HEC. (**a**) HEC is rehydrated with crystallization buffer in 500 ml Hamilton syringe. (**b**) The required amount of cellulose is transferred in a 100 ml Hamilton syringe. (**c**) LOV crystals in solution are inserted at the back of a 100 ml Hamilton syringe with caution to avoid bubbles. (**d**) LOV crystals are mixed with the cellulose using a three-way coupler until homogeneity. (**e**) Cellulose embedded crystals are then loaded in the reservoir of the HVE injector.

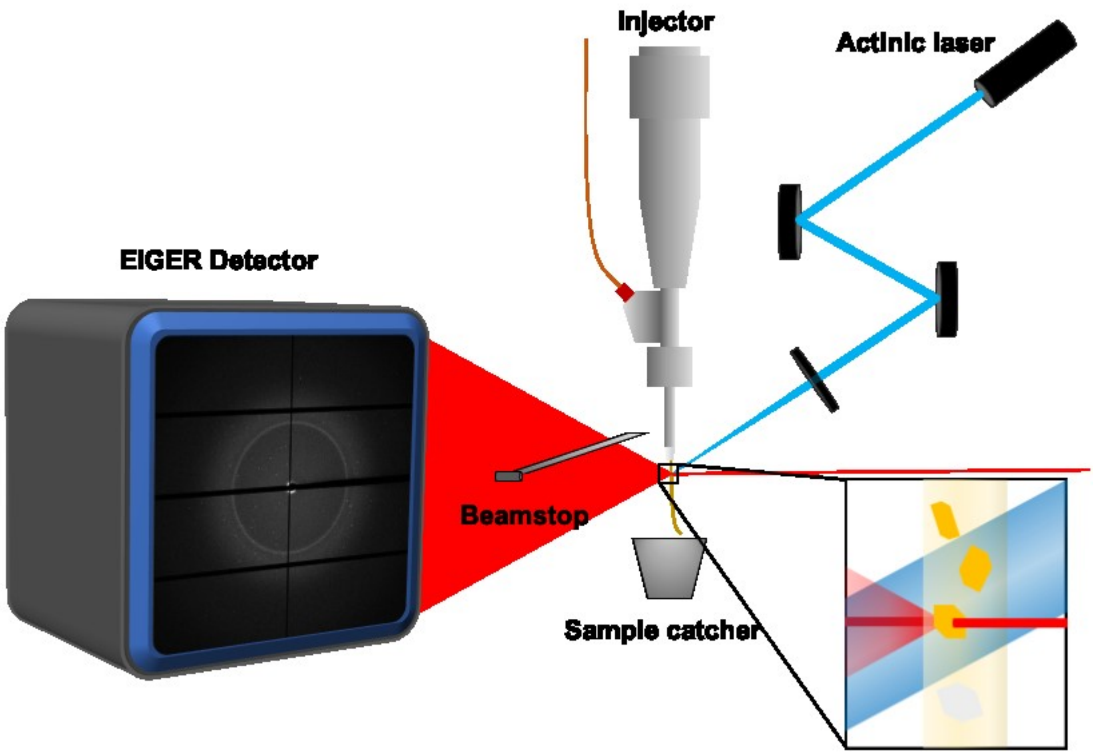

Fig. S4 Time resolved serial synchrotron crystallography setup. LOV crystals are serially injected onto the path of the X-ray synchrotron beam. Crystals are photoexcited using a 470 nm focused laser that is synchronized with the trigger of the detector. Diffraction patterns are collected following the defined data collection scheme (**Fig. S5**).

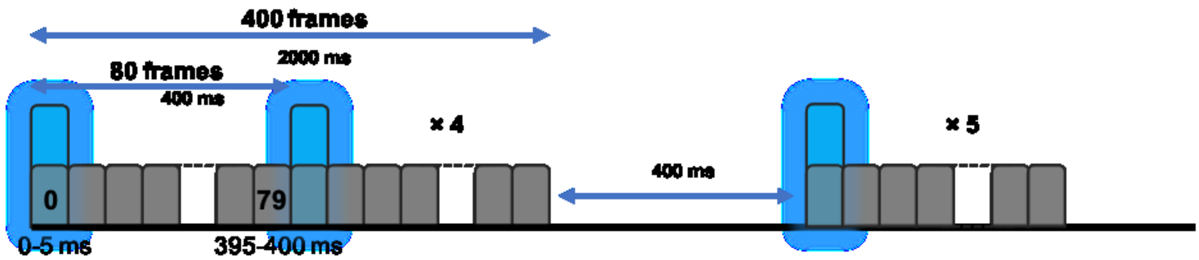

Fig. S5 Schematic representation of the pulse / detector organization for the TR-SSX data collection on LOV1. The activation sequence is composed of one 5 ms frame collected with the laser diode on (blue histogram), followed by 79 frames collected without illumination (gray histogram). The sequence is repeated 5 times, after which one activation sequence is skipped.

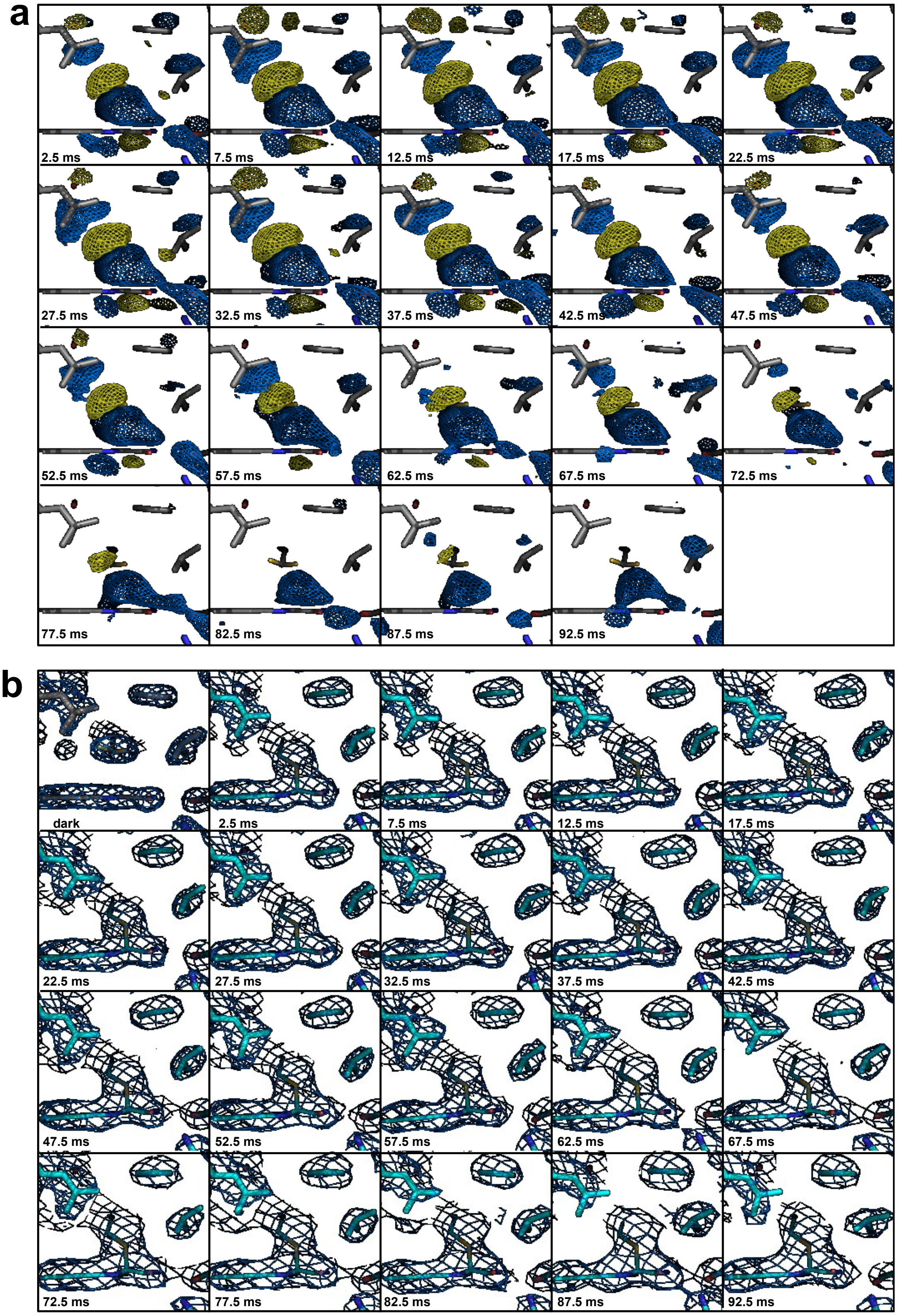

Fig. S6 Fourier difference and extrapolated electron density maps. (**a**) $F_{obs}^{light(n)} - F_{obs}^{dark}$ from 2.5 to 92.5 ms after photoactivation surrounding the adduct represented at ±3.0 σ showing the slow decrease signal in the maps with positive density (blue) and negative density (gold) shown as mesh.

(**b**) Dark state $2F_{obs} - F_{calc}$ shown as dark blue mesh around the flavin site with protein model shown as gray sticks and $2F_{ext} - F_{calc}$ extrapolated maps from 2.5 to 92.5 ms shown as blue mesh with protein model is shown as cyan sticks.

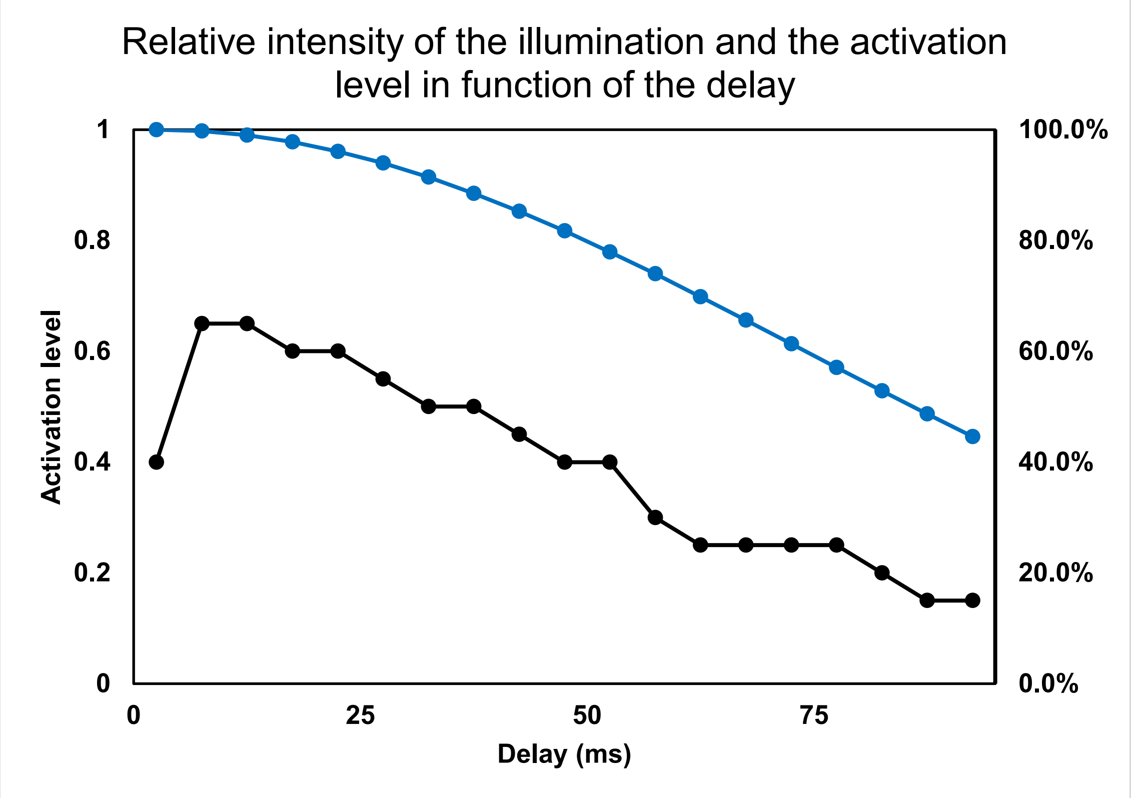

Fig. S7 Relative intensity of the illumination (blue) and of the activation level (black) as function of the probed delay, which display a common trend.

Supplementary Scripts

The supplementary scripts include scripts used to execute processing of the data in this work. The zipped folder includes a README file with detailed description of their purpose and use.

References

Arinkin, V., Granzin, J., Krauss, U., Jaeger, K., Willbold, D. & Batra-Safferling, R. (2021). *FEBS J* **288**, 4955–4972.

Arinkin, V., Granzin, J., Röllen, K., Krauss, U., Jaeger, K.-E., Willbold, D. & Batra-Safferling, R. (2017). *Sci Rep* **7**, 42971.

Aumonier, S., Santoni, G., Gotthard, G., von Stetten, D., Leonard, G. A. & Royant, A. (2020). *IUCrJ* **7**, 728–736.

Banerjee, A., Herman, E., Kottke, T. & Essen, L. O. (2016). *Structure* **24**, 171–178.

Christie, J. M., Hitomi, K., Arvai, A. S., Hartfield, K. A., Mettlen, M., Pratt, A. J., Tainer, J. A. & Getzoff, E. D. (2012). *Journal of Biological Chemistry* **287**, 22295–22304.

Circolone, F., Granzin, J., Jentzsch, K., Drepper, T., Jaeger, K. E., Willbold, D., Krauss, U. & Batra-Safferling, R. (2012). *J Mol Biol* **417**, 362–374.

Conrad, K. S., Bilwes, A. M. & Crane, B. R. (2013). *Biochemistry* **52**, 378–391.

Crosson, S. & Moffat, K. (2001). *Proceedings of the National Academy of Sciences* **98**, 2995–3000.

Crosson, S. & Moffat, K. (2002). *Plant Cell* **14**, 1067–1075.

Diensthuber, R. P., Bommer, M., Gleichmann, T. & Möglich, A. (2013). *Structure* **21**, 1127–1136.

Dietler, J., Schubert, R., Krafft, T. G. A., Meiler, S., Kainrath, S., Richter, F., Schweimer, K., Weyand, M., Janovjak, H. & Möglich, A. (2021). *J Mol Biol* **433**, 167107.

Endres, S., Granzin, J., Circolone, F., Stadler, A., Krauss, U., Drepper, T., Svensson, V., Knieps-Grünhagen, E., Wirtz, A., Cousin, A., Tielen, P., Willbold, D., Jaeger, K. E. & Batra-Safferling, R. (2015). *BMC Microbiol* **15**, https://doi.org/10.1186/s12866-015-0365-0.

Fedorov, R., Schlichting, I., Hartmann, E., Domratcheva, T., Fuhrmann, M. & Hegemann, P. (2003). *Biophys J* **84**, 2474–2482.

Fettweiss, T., Röllen, K., Granzin, J., Reiners, O., Endres, S., Drepper, T., Willbold, D., Jaeger, K. E., Batra-Safferling, R. & Krauss, U. (2018). *Biochemistry* **57**, 4833–4847.

Goncharov, I. M., Smolentseva, A., Semenov, O., Natarov, I., Nazarenko, V. V., Yudenko, A., Remeeva, A. & Gushchin, I. (2021). *Biochem Biophys Res Commun* **567**, 143–147.

Gotthard, G., Aumonier, S., De Sanctis, D., Leonard, G., von Stetten, D. & Royant, A. (2019). *IUCrJ* **6**, 665–680.

Halavaty, A. S. & Moffat, K. (2007). *Biochemistry* **46**, 14001–14009.

Halavaty, A. S. & Moffat, K. (2013). *Acta Crystallogr Sect F Struct Biol Cryst Commun* **69**, 1316–1321.

Heintz, U. & Schlichting, I. (2016). *Elife* **5**, 1–21.

Hepp, S., Trauth, J., Hasenjäger, S., Bezold, F., Essen, L. O. & Taxis, C. (2020). *J Mol Biol* 1880–1900.

Kalvaitis, M. E., Johnson, L. A., Mart, R. J., Rizkallah, P. & Allemann, R. K. (2019). *Biochemistry* **58**, 2608–2616.

Key, J., Hefti, M., Purcell, E. B. & Moffat, K. (2007). *Biochemistry* **46**, 3614–3623.

Lamb, J. S., Zoltowski, B. D., Pabit, S. A., Li, L., Crane, B. R. & Pollack, L. (2009). *J Mol Biol* **393**, 909–919.

Lokhandwala, J., Hopkins, H. C., Rodriguez-Iglesias, A., Dattenböck, C., Schmoll, M. & Zoltowski, B. D. (2015). *Structure* **23**, 116–125.

Mitra, D., Yang, X. & Moffat, K. (2012). *Structure* **20**, 698–706.

Möglich, A. & Moffat, K. (2007). *J Mol Biol* **373**, 112–126.

Nakasako, M., Zikihara, K., Matsuoka, D., Katsura, H. & Tokutomi, S. (2008). *J Mol Biol* **381**, 718–733.

Nash, A. I., McNulty, R., Shillito, M. E., Swartz, T. E., Bogomolni, R. A., Luecke, H. & Gardner, K. H. (2011). *Proc Natl Acad Sci U S A* **108**, 9449–9454.

Nazarenko, V. V., Remeeva, A., Yudenko, A., Kovalev, K., Dubenko, A., Goncharov, I. M., Kuzmichev, P., Rogachev, A. V., Buslaev, P., Borshchevskiy, V., Mishin, A., Dhoke, G. V., Schwaneberg, U., Davari, M. D., Jaeger, K.-E., Krauss, U., Gordeliy, V. & Gushchin, I. (2019). *Photochemical & Photobiological Sciences* **18**, 1793–1805.

Pudasaini, A., Green, R., Song, Y. H., Blumenfeld, A., Karki, N., Imaizumi, T. & Zoltowski, B. D. (2021). *Biochemistry* **60**, 95–103.

Pudasaini, A., Shim, J. S., Song, Y. H., Shi, H., Kiba, T., Somers, D. E., Imaizumi, T. & Zoltowski, B. D. (2017). *Elife* **6**, 1–27.

Remeeva, A., Nazarenko, V. V, Goncharov, I. M., Yudenko, A., Smolentseva, A., Semenov, O., Kovalev, K., Gülbahar, C., Schwaneberg, U., Davari, M. D., Gordeliy, V. & Gushchin, I. (2020). *Crystals (Basel)* **10**, 256.

Remeeva, A., Nazarenko, V. V., Kovalev, K., Goncharov, I. M., Yudenko, A., Astashkin, R., Gordeliy, V. & Gushchin, I. (2021). *Proteins: Structure, Function, and Bioinformatics* **89**, 1005–1016.

Rinaldi, J., Fernández, I., Shin, H., Sycz, G., Gunawardana, S., Kumarapperuma, I., Paz, J. M., Otero, L. H., Cerutti, M. L., Zorreguieta, Á., Ren, Z., Klinke, S., Yang, X. & Goldbaum, F. A. (2021). *MBio* **12**, 1–18.

Rinaldi, J., Gallo, M., Klinke, S., Paris, G., Bonomi, H. R., Bogomolni, R. A., Cicero, D. O. & Goldbaum, F. A. (2012). *J Mol Biol* **420**, 112–127.

Rivera-Cancel, G., Ko, W., Tomchick, D. R., Correa, F. & Gardner, K. H. (2014). *Proceedings of the National Academy of Sciences* **111**, 17839–17844.

Röllen, K., Granzin, J., Panwalkar, V., Arinkin, V., Rani, R., Hartmann, R., Krauss, U., Jaeger, K. E., Willbold, D. & Batra-Safferling, R. (2016). *J Mol Biol* **428**, 3721–3736.

Röllen, K., Granzin, J., Remeeva, A., Davari, M. D., Gensch, T., Nazarenko, V. V., Kovalev, K., Bogorodskiy, A., Borshchevskiy, V., Hemmer, S., Schwaneberg, U., Gordeliy, V., Jaeger, K. E.,

Batra-Safferling, R., Gushchin, I. & Krauss, U. (2021). *Journal of Biological Chemistry* **296**, 100662.

Vaidya, A. T., Chen, C. H., Dunlap, J. C., Loros, J. J. & Crane, B. R. (2011). *Sci Signal* **4**, 1–8.

Wang, H., Vilela, M., Winkler, A., Tarnawski, M., Schlichting, I., Yumerefendi, H., Kuhlman, B., Liu, R., Danuser, G. & Hahn, K. M. (2016). *Nat Methods* **13**, 755–758.

Wu, Y. I., Frey, D., Lungu, O. I., Jaehrig, A., Schlichting, I., Kuhlman, B. & Hahn, K. M. (2009). *Nature* **461**, 104–108.

Zoltowski, B. D. & Crane, B. R. (2008). *Biochemistry* **47**, 7012–7019.

Zoltowski, B. D., Schwerdtfeger, C., Widom, J., Loros, J. J., Bilwes, A. M., Dunlap, J. C. & Crane, B. R. (2007). *Science (1979)* **316**, 1054–1057.

Zoltowski, B. D., Vaccaro, B. & Crane, B. R. (2009). *Nat Chem Biol* **5**, 827–834.
